# Literary Notes

**Published:** 1897-12

**Authors:** 


### Literary Notes.

THE *Philadelphia Medical Journal* is the name of a new weekly, to begin in January, 1898, under the editorship of Dr. George M. Gould. It is owned by a stock company and published by a board of trustees. Philadelphia needs a weekly medical journal and Dr. Gould is just the man to edit it.

---

THE Medical News Visiting List for 1898 has made its appearance. It is issued in several forms, as usual—namely, weekly, dated for 30 patients; monthly, undated, for 120 patients per month; perpetual, undated, for 30 patients weekly per year and perpetual, undated, for 60 patients weekly per year. The first three styles contain 32 pages of data and 160 pages of blanks. The 60-patient perpetual consists of 256 pages of blanks. Each style in one wallet-shaped book, with pocket, pencil and rubber. Seal grain leather, $1.25 ; thumb letter index, 25 cents extra.

The book begins with 32 pages of printed data of the most useful sort, including an alphabetical table of diseases with approved remedies, a table of doses, sections on examination of urine, artificial respiration, incompatibles, poisons and antidotes, a diagnostic table of eruptive fevers and a full-page plate, showing at a glance the incisions for ligation of the various arteries, an invaluable guide in such emergencies.

It goes without saying that a visiting list is an indispensable part of the equipment of an active physician. This is one of the best of its kind and can be obtained by addressing the publishers, Messrs. Lea Brothers & Company, 706 Sansom street, Philadelphia, or 111 Fifth avenue, New York.

---

The Columbia Calendar for 1898 has come to hand. This is its thirteenth year, and its general style is the same as formerly. It contains a convenient arrangement of dates that will prove useful to busy men, and as plenty of space is reserved for memoranda, the pad may be used as a diary and as a reminder for business appointments and obligations. It is neat in appearance, takes up but little room and is both ornamental and useful for the desk, while its stand is of such character that it may be used either upon the desk or hung upon the wall.

The moon's phases are indicated in the calendar for the benefit of those who wish to have this information. The calendar is ready for distribution and all orders for it will be filled upon the day of receipt. It can be obtained by mail, prepaid, for five two-cent stamps by addressing the calendar department of the Pope Manufacturing Company, Hartford, Conn.

---

The *Chicago Clinic* is the name of the journal that was formerly known as the *Omaha Clinic.* It has become the organ of the Chicago Clinical School (formerly the West-side Post-Graduate School and Polyclinic).

Dr. J. Homer Coulter will remain as editor and manager of *The Clinic* under its new name and form. Address 819-823 W. Harrison street.

